# Patients’ Experiences with and Attitudes towards a Diabetes Patient Web Portal

**DOI:** 10.1371/journal.pone.0129403

**Published:** 2015-06-18

**Authors:** Maaike C. M. Ronda, Lioe-Ting Dijkhorst-Oei, Guy E. H. M. Rutten

**Affiliations:** 1 Julius Centre for Health Sciences and Primary Care, University Medical Center, Utrecht, the Netherlands; 2 Department of Internal Medicine, Meander Medical Center, Amersfoort, the Netherlands; Iran University of Medical Sciences, IRAN, ISLAMIC REPUBLIC OF IRAN

## Abstract

**Objective:**

A diabetes patient web portal allows patients to access their personal health record and may improve diabetes outcomes; however, patients’ adoption is slow. We aimed to get insight into patients’ experiences with a web portal to understand how the portal is being used, how patients perceive the content of the portal and to assess whether redesign of the portal might be needed.

**Materials and Methods:**

A survey among 1500 patients with type 1 and type 2 diabetes with a login to a patient portal. Setting: 62 primary care practices and one outpatient hospital clinic, using a combined patient portal. We compared patients who requested a login but never used it or once (‘early quitters’) with patients who used it at least two times (‘persistent users’).

**Results:**

632 patients (42.1%) returned the questionnaire. Their mean age was 59.7 years, 63.1% was male and 81.8% had type 2 diabetes. 413 (65.3%) people were persistent users and 34.7% early quitters. In the multivariable analysis, insulin use (OR2.07; 95%CI[1.18–3.62]), experiencing more frequently hyperglycemic episodes (OR1.30;95%CI[1.14–1.49]) and better diabetes knowledge (OR1.02, 95%CI[1.01–1.03]) do increase the odds of being a persistent user. Persistent users perceived the usefulness of the patient portal significantly more favorable. However, they also more decisively declared that the patient portal is not helpful in supporting life style changes. Early quitters felt significantly more items not applicable in their situation compared to persistent users. Both persistent users (69.8%) and early quitters (58.8%) would prefer a reminder function for scheduled visits. About 60% of both groups wanted information about medication and side-effects in their portal.

**Conclusions:**

The diabetes patient web portal might be improved significantly by taking into account the patients’ experiences and attitudes. We propose creating separate portals for patients on insulin or not.

## Introduction

A patient web portal (PWP) can help patients increase their knowledge about the disease [1], improve diabetes outcomes [2–6], increase self-efficacy [7] and getting patients more involved in their own treatment [8]. However, adoption rates to web portals are slow. Our group and others have found differences between users and non-users of a diabetes web portal on both demographic and diabetes related variables [9,10]. Health care providers need to focus on these differences and give extra attention to patients who could benefit from portal use. We also need to examine the way patients use a web portal and to gain insight into a patient’s perspective of the usefulness of a PWP to increase its use.

Patients start using a PWP to increase their self-management [7], to enhance the communication with their health care provider [11] or because of dissatisfaction with the patient-provider relationship [12]. There are barriers that prevent patients from starting or continuing the use of a web portal, such as fear for privacy [13], non-feedback frustration and difficulty implementing PWP use in daily life [14]. Some patients may have incorrect assumptions about a PWP leading to expectations that are not met [15]. Furthermore, patients have specific wishes for content and additional personalized online services to improve portals [13].

Because many portals have been designed by physicians and IT-specialists, and not by patients themselves, redesign of the web portals might be needed to interest as many patients as possible and to address their specific wishes and needs. We aimed to gain insight into the experiences, motivations and preferences of persistent users and early quitters of a diabetes PWP.

The following research questions were addressed: 1. What are the characteristics of patients who request a login and become a persistent user in comparison to patients who cease to use the portal in an early stage? 2. Why do patients request a login to the web portal? 3. How is the web portal being used? 4. How do patients assess the content of the web portal? 5. What are the patients’ wishes for improvement?

## Materials and Methods

### Study setting and design

All participants had to sign a consent form to participate. In the Netherlands, studies involving human subjects need to undergo a medical ethics review if they are subjected to the Medical Research Involving Human Subject Act (WMO). This study was assessed and considered non-WMO applicable by the Medical Ethics Committee of the University of Utrecht, which means that no further ethical approval was required (protocol number 11-296/C).

**Fig 1:**
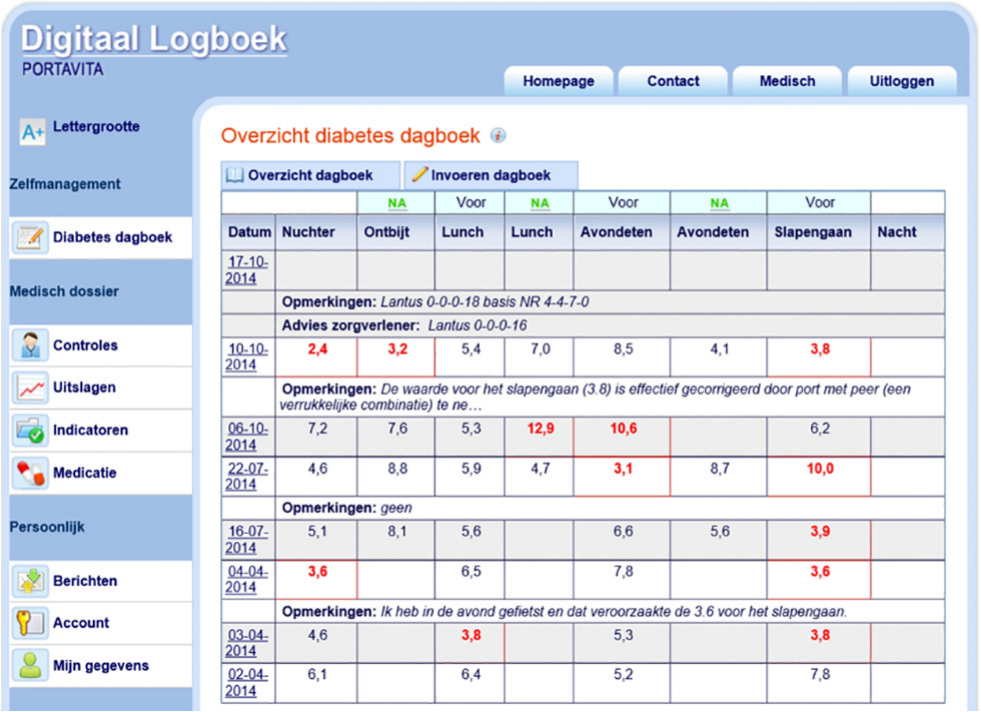
Screenshot of the glucose diary of the patient portal.

‘Diamuraal’ is an organization that coordinates the diabetes care in a defined geographical area in the center of the Netherlands. It comprises 62 independent primary care practices and one outpatient clinic of the regional hospital that provide diabetes care to over 10.000 patients, working in a care group [16,17]. All physicians and nurses who participate in the care of these patients record their data in the same electronic health record and patients can request a log-in to access their personal medical records. This portal is called ‘Digitaal Logboek’ and was developed by Diamuraal and a private company (Portavita). After login, patients have access to the information provided by their physician or nurse during medical consultations. These include full-text of the clinic notes, the results of physical examination, laboratory results, problem lists and treatment goals. Patients can view a list of their current use of medications, however the completeness of this list is depending on the physician because this needs to be manually added. The PWP also provides general diabetes information and an overview of all examinations and visits that are needed in high quality diabetes care. Patients can upload glucose levels measured at home (Fig 1) and contact their personal care provider through secured e-messaging. This portal is an integral part of the EMR, all interactions and messages between patient and provider are stored in the EMR. The portal is additional; patients who have not requested access receive usual diabetes care. We conducted a survey among adult patients with type 1 or 2 diabetes mellitus. The physicians working within the organization of ‘Diamuraal’ have registered their patients with this organization and all data about patient characteristics and data concerning the disease are recorded in the electronic health record, including if a patient has requested a login to the web portal. For this survey we randomly selected 1500 patients aged 18–85 years with a login to the web portal. As part of our study, we also sent different questionnaires to patients within ‘Diamuraal’ that are registered as not having a login (non-users). Information about the latter group of patients has been published elsewhere and is beyond the scope of this paper [18]. Patients were sent an informational letter together with a questionnaire. They received a reminder twice in a three week interval. All participants had to sign a consent form to participate. In the Netherlands, studies involving human subjects need to undergo a medical ethics review if they are subjected to the Medical Research Involving Human Subject Act (WMO). This study was assessed and considered non-WMO applicable by the Medical Ethics Committee of the University of Utrecht, which means that no further ethical approval was required (protocol number 11-296/C).

### Study measures

We collected patient data form the electronic health record, such as login frequency, age, gender, type of diabetes, treatment setting, laboratory values, comorbidity and diabetic complications.

The questionnaire about the portal contained multiple choice questions about reasons for requesting a login, the usability of portal features and patient’s wishes. For all questions, see S1 Questionnaire. There were three questions that were scored on a 5-point Likert scale. We combined the two highest categories (very useful and useful; very satisfied and satisfied; very important and important) in the analysis.

We used an additional small questionnaire for asking educational level, ethnicity, living status, employment, medication, current smoking, drinking alcohol, physical activity, access to internet and to a computer. Finally we added several validated questionnaires to measure: satisfaction with diabetes treatment (The Diabetes Treatment Satisfaction Questionnaire, DTSQ) [19]; diabetes-specific distress (Problem Areas in Diabetes, PAID) [20,21]; self-efficacy (Diabetes Management Self-Efficacy Scale, DMSES) [22] and diabetes knowledge (Brief Diabetes Knowledge Test, BDKT) [23,24]. These measures are described in more detail elsewhere [9]. Data is deposited in Dryad [25].

### Statistical analysis

The patients were analyzed according to their login-frequency. We compared two groups: patients who requested a login but never used it or only once (‘early quitters’) and patients who requested a login and used it at least two times (‘persistent users’). This division is based on registered data on the number of actual logins in the patient web portal from the first access to the portal. The period of access may range from about three years to just a few months. Our definition of persistent users and early quitters is comparable to data other studies ([26,27]).

Categorical variables were expressed as percentages and continuous variables as means with standard deviation (SD). We used χ2-tests for all categorical variables and unpaired t-tests for all continuous variables. Logistic regression was used to determine which variables are independently associated with the cessation or continuation of the portal. We used a p-value of <0.2 in the univariable analysis to select variables for multivariable analysis. The reasons for use, the answers about content and usefulness of the portal and about the wishes were expressed as percentages. The answer categories ‘useful’ and ‘very useful’ were combined.

Data was analyzed using SPSS for Windows (versions 20, SPSS Inc., Chicago, IL, USA).

## Results

From 1500 questionnaires 24 were undeliverable. Of the 1476 patients who received a questionnaire, 632 (42.8%) patients returned a completed questionnaire and were eligible for analysis (responders). Their mean age was 59.7 ± 13.2 years (versus non-responders 56.8 ± 15.1, p<0.001) and 63.1% was male (versus non-responders 57.1%, p = 0.02). 413 (65.3%) patients were ‘persistent users’ (PU) and 219 (34.7%) patients were ‘early quitters’ (EQ).

### Characteristics of early quitters versus persistent users

Persistent users were younger and had more often a paid job. More of them used insulin, were treated by an internist and used the internet daily. They had better diabetes knowledge and experienced both more hypoglycemic and hyperglycemic episodes (Table 1). The use of insulin, more frequently perceived hyperglycemic episodes and better diabetes knowledge increased the odds of becoming a persistent user. With a higher HbA1c the odds of becoming a persistent users decreases (Table 2). When using the internet, responders from both groups were mostly over an hour online (PU 45.4% versus EQ 36.0%, p = 0.18). Furthermore, 51.1% of the persistent users declared that they used the internet for searching information about their disease compared with only 22.0% of the early quitters (p<0.001).

**Table 1:** Characteristics of the study participants (n = 632), mean ± SD or %.

		Early quitters (n = 219)	Persistent users (n = 413)	P-value
Age, years		61.9 ± 12.7	58.5 ± 13.3	0.02
Gender, male		63.9	62.7	0.76
Caucasian		91.2	93.6	0.26
Educational level, high		39.4	46.2	0.11
Paid job		36.3	47.1	0.01
Living arrangement, alone		16.8	15.9	0.76
Fluency in speaking Dutch		97.2	99.3	0.07
Daily use of internet		63.0	77.9	<0.001
Treatment setting	General practitioner	54.8	43.6	0.01
	Internist	45.2	56.4	
Type of Diabetes	Type 1	15.5	19.6	0.21
	Type 2	84.5	80.4	
Duration of Diabetes, years		13.9 ± 11.0	13.3 ± 10.7	0.49
Insulin		45.9	63.2	< 0.001
Polypharmacy*		47.2	52.7	0.21
HbA1c (mmol/mol)		54.0 ± 12.0	55.5 ± 11.2	0.14
Total cholesterol (mmol)		4.3 ± 1.0	4.4 ± 1.0	0.46
Current smoker		16.4	11.5	0.09
Drinking alcohol		49.3	52.8	0.41
PAID (range 0–100)		29.3 ± 11.5	31.0 ± 11.8	0.11
DMSES (range 20–100)		80.7 ± 16.5	80.7 ± 15.5	0.97
BDKT standard (range 0–100)		70.6 ± 18.8	78.7 ± 14.7	< 0.001
BDKT insulin (range 0–100)		58.0 ± 19.2	61.4 ± 20.6	0.15
DTSQ status (range 0–36)		29.8 ± 5.3	30.2 ± 5.0	0.37
DTSQ hyper (range 0–6)		2.0 ± 1.8	2.7 ± 1.9	< 0.001
DTSQ hypo (range 0–6)		1.6 ± 1.6	2.0 ± 1.7	0.01

* polypharmacy: the use of five or more medications.

**Table 2:** Independent determinants of becoming regular users compared to early quitters.

	OR (95% CI)	P-value
Insulin	2.07 (1.18–3.62)	0.01
HbA1c	0.97 (0.95–0.99)	< 0.01
BDKT standard	1.02 (1.01–1.03)	< 0.01
DTSQ hyper	1.30 (1.14–1.49)	<0.001

### Reasons for requesting a login

The majority of patients from both groups declared that they ‘discovered’ the existence of the PWP after being informed by their physician (PU 94.9%, EQ 77.6%, p<0.001). For persistent users, the main two reasons for requesting a login were that the portal could give them access to the laboratory results and treatment goals (75.5%) and that the portal could influence disease and management (42.5%). For early quitters, the two main reasons for requesting a login were the access to the clinic notes and laboratory results (42.9%) and the suggested use of the portal by others (20.5%).

### The general usefulness and usefulness of specific content

The majority of the persistent users (53.1%) accessed the web portal less than once a month and half of them spent less than fifteen minutes per session. They declared it easy to use (PU 91.9% versus EQ 78.7%, p<0.001); easy to login (PU 96.8% versus EQ 86.0%, p<0.001); they were satisfied with the layout (PU 96.8% versus EQ 85.2%, p<0.001) and assessed the overall information to be comprehensible (PU 97.5% versus EQ 90.4%, p = 0.01). The same held true for the comprehensibility of specific web portal items: the meaning of laboratory values (PU 92.0% versus EQ 77.1%, p<0.001), the abbreviations used (PU 75.8% versus EQ 54.9%, p<0.001), the medical phrasings (PU 69.4% versus EQ 49.0%), p<0.001) and the reasons of why the appointments and check-ups in the clinic are needed (PU 91.7% versus EQ 73.0%, p<0.001). The majority of both persistent users (77.0%) and early quitters (79.3%) declared that they never had contacted the helpdesk for support (p = 0.66). Of the people who did contact the helpdesk the main reason in both groups was because of losing their passwords (PU 49.5% versus EQ 64.7%, p = 0.28).

Both persistent users and early quitters appreciated most that they could reread at their homes the information discussed during consultations, the access to their laboratory values and treatment goals; persistent users rated the usefulness of all these items significantly higher than early quitters (Table 3).

**Table 3:** Early quitters and persistent users regarding the perceived usefulness (very useful or useful) of the content items of the patient web portal.

	Early quitters (n = 219)		Persistent users (n = 413)		P-value
	n*	% agree	n*	% agree	
Summary of upcoming visits	147	65.3	401	78.8	< 0.01
Summary of all physicians / caregivers	144	52.8	396	61.4	0.18
e-messaging	144	56.2	401	74.6	< 0.001
General diabetes information	144	42.4	396	53.8	0.06
Glucose diary	144	47.2	401	72.1	< 0.001
Rereading clinic visit	146	72.6	402	89.6	< 0.001
Laboratory values + treatment goals	147	72.1	403	92.3	< 0.001
Summary of all controls (past and future)	146	67.8	402	84.1	<0.001
Summary of medication	144	62.5	401	64.6	0.90

* number of patients who answered that question.

More PU than EQ stated that they know their own HbA1c and cholesterol levels and the targets for weight, HbA1c and blood pressure. When asked if the portal helps with supporting life style changes, about half of PU scored items negatively. The EQ felt significantly more items not applicable in their situation compared to PU (Table 4).

**Table 4:** Opinions of early quitters (n = 219) versus persistent users (n = 413) about the way the portal being supportive for care.

Survey question	Early quitters				Persistent users				P-value
	n*	% Yes	% No	% n/a	n*	% Yes	% No	% n/a	
**Do you know…?**									
…the value of your own weight?	189	100			409	99.3			0.24
…the value of your blood pressure?	184	94.6			407	93.1			0.68
…the value of your Hba1c?	184	60.3			402	82.1			<0.001
… the value of your cholesterol?	184	70.7			408	85.3			<0.001
…the treatment goals of your weight?	183	88.0			407	92.9			0.05
…the treatment goals of your blood pressure?	183	84.6			407	91.9			<0.01
…the treatment goals of your HbA1c?	179	62.0			402	82.6			<0.001
…the treatment goals of your weight?	178	96.1			404	83.7			<0.001
**Do you believe the portal will help with…?**									
…adherence to diet	165	14.5	56.4	29.1	405	15.6	66.7	17.8	0.01
…adherence to sport	166	10.2	56.0	33.7	402	11.4	66.7	21.9	0.01
…losing weight	167	17.4	53.9	28.7	401	17.5	60.3	22.2	0.23
…stop smoking	168	4.2	40.5	55.4	401	3.7	37.4	58.9	0.83
…adherence in taking medication	166	15.7	51.2	33.1	393	21.4	64.1	14.5	<0.001
…diabetes knowledge	166	34.9	39.2	25.9	401	49.4	43.4	7.5	<0.001
…preventing complications	167	21.6	49.1	29.3	403	32.8	55.8	11.4	<0.001

* number of patients who gave an answer to that question.

### Wishes for improvement

Persistent users and early quitters answer differently about additional items which could improve the web portal (Table 5). PU want to be able to add their injected insulin units to the glucose diary, to receive updates with current medical information about diabetes and to use the portal for supporting the diabetes care, like scheduling a clinic visit. Among EQ the desires concerning reminder functions for upcoming visits, information about medication and side-effects and automatic upload from glucose meters are most often listed. It should be noted that the majority of PU also wish to have these functionalities added to the PWP.

**Table 5:** Wishes about additional functionalities.

	Early quitters (n = 219)		Persistent users (n = 413)		P-value
	n*	% agree	n*	% agree	
Automatic signal to physician by uploading glucose diary	164	74 (45.1)	402	197 (49.0)	0.48
Automatic upload from glucose meter to portal	162	85 (52.5)	399	231 (57.9)	0.49
Adding insulin units to glucose diary	118	60 (50.8)	297	199 (67.0)	< 0.001
Links to websites with information about diabetes	170	43 (25.3)	397	167 (42.1)	< 0.01
Links to websites with interventions	167	40 (24.0)	394	111 (28.2)	0.58
Portal on mobile device	166	21 (12.7)	393	78 (19.8)	0.10
Request for medication refills	168	84 (50.0)	396	231 (58.3)	0.11
Forum	165	25 (15.2)	396	70 (17.7)	0.69
Printing functionality	163	70 (42.9)	396	197 (49.7)	0.23
Updates with current medical information about diabetes	167	86 (51.5)	395	245 (61.7)	0.01
Information in different languages	166	18 (10.1)	393	31 (7.9)	0.26
Information about medication and side effects	169	95 (56.2)	401	238 (59.4)	0.75
Reminder function when scheduled / upcoming visit is due	170	100 (58.8)	404	282 (69.8)	0.04
Using the portal for scheduling a visit with physician	170	73 (42.9)	403	263 (65.3)	< 0.001

* number of patients who answered that question.

## Discussion

This study provides insight into the experiences, motivations and preferences of persistent users and early quitters of a diabetes web portal. With this information we can adjust the portal to the potential users’ wishes and preferences.

The main reason all patients requested access to the patient web portal was because it could give them access to laboratory results and treatment goals. Apparently patients are interested in using a PWP as a tool in managing their disease. Most patients ‘discovered’ the existence of the PWP after being informed by their care provider. Among the EQ there was a large group that got interested in the PWP by other means like posters in waiting areas or pamphlets. We assume that most of the latter group did not discuss the PWP with their treating physician. This implies that there is an important role for the health care provider in turning the patient into a PU. Modelling expectations can prevent early quitting due to disappointment [15]. Referral to the PWP during consultations may prompt patients to return to their PWP.

Patients who became persistent users were apparently those with a higher disease seriousness. Also among parents with children with a chronic disease, low level of disease severity was one of the reasons for not using the portal [28]. However, a recent systematic review on the use of electronic portal usage among patients with diabetes showed mixed outcomes in this respect [29]. We may conclude that one uniform portal is not suitable for all patients and we should consider dividing a diabetes web portal immediately after the entrance in two parts: one for patients who are injecting insulin and another for patients who do not. In patients with type 2 diabetes from the same organization, we previously found insulin use is a predictor of requesting a login [9]. Designing different portals will meet the needs of different categories of patients and could also meet the preferences of early quitters to get more information about (oral) medication and its side-effects.

Even two thirds of the persistent users responded that they did not feel the portal supports them in most lifestyle choices. We do not know the reason for this, but it might be because in the current portal most of these items are incorporated in other parts of the portal, like in the free text box at the end of the consult summary. In redesigning the portal, this finding has to be taken into account. A second explanation could be, that most of the users have both a low frequency and a low duration of accessing the portal, as in other studies [28,30]. This low frequency could explain why patients consider it not supportive in incorporating its information about life-style changes in daily life [14]. We could help reminding the patients using the PWP by a simple adjustment in portal functionality, e.g. an automatically generated email to remind patients to log in and evaluate their lifestyle and the agreement they made about it with their physician. If necessary they can use the e-messaging for questions and support when encountering difficulties in the implementation. In other types of web portals, weight and activity logs are implemented to encourage life style changes [11].

Persistent users perceived the comprehensibility of the portal more favorable than early quitters. One of the reasons of early quitting might be the medical language. Indeed, medical terms and abbreviations require explanation [13,31]. Besides the already available online manual we could offer a course or workshop on navigating through and understanding the portal. The ideas we offer in this paper for improvement of the patient web portal are against the background that the PWP we studied is a static coded website. Other portals might use technology that allows a more dynamic approach, in which sections appear based on patient characteristics. For new portals that are still in a design phase, this should be taken into consideration.

Study strengths include a large and representative population with both type 1 and type 2 diabetes patients and patients from primary as well as secondary care. The diabetes portal in this study is already 6 years in use, which adds to the value of patients’ opinions. Furthermore, besides the survey data about users’ opinions we used actual data about number of logins and patient characteristics, derived from the central ‘Diamuraal’ database, that encompasses all patients with diabetes mellitus treated by primary care physicians and internists who participate in ‘Diamuraal’.

Nevertheless, there are limitations: only 42.8% of the approached people responded. This percentage is comparable with a previously found willingness of diabetes patients in participating in research [32]. Our participants were slightly older and more frequently male. However, both age and gender were not a determinant for becoming a persistent user; therefore the selective participation may not have influenced the outcomes. It is unclear if we can generalize our results to the entire diabetes population in the Netherlands because there is no national diabetes registry. However, irrespective of the representativeness of our study population, issues raised in this paper about problems with comprehensibility of the portal, supporting lifestyle changes and additional wishes for portal features should be taken into account when designing a patient portal for patients with diabetes. Another limitation is the cut-off point of 2 times login for the definition ‘persistent user’ or ‘early quitter’. To the best of our knowledge there is no definition of how many login times makes a person a persistent user. For that reason we had to make a judgment call based on the distribution of actual logins from the first access to the portal. This paper does not include information about the group of patients that never requested a login (the so called ‘non-users’). They are not able to provide information about the use of the portal the scope of this paper. Compared to users, the non-users are older (59.7±13.2 years vs. 67.4±10.0 years, P<0.001) and less frequently male (63.1% vs. 56.6%).

In conclusion, medical terms and abbreviations in a PWP require explanation. Patients who are prescribed insulin, perceive hypoglycemic episodes and have better diabetes knowledge are the ones who become persistent users of a PWP. Persistent users evaluate the portal more favorable and would like to be able to add their injected insulin units to the glucose diary. We consider dividing a diabetes web portal immediately after the entrance in two parts: one for patients who are injecting insulin and another for patients who do not. This suggestion also meets the preferences of early quitters to get more information about (oral) medication and its side-effects.

## Supporting Information

S1 Questionnaire (DOCX)
