# Correction: Patients’ Experiences with and Attitudes towards a Diabetes Patient Web Portal

**DOI:** 10.1371/journal.pone.0133572

**Published:** 2015-07-20

**Authors:** Maaike C. M. Ronda, Lioe-Ting Dijkhorst-Oei, Guy E. H. M. Rutten

The Data Availability Statement appearing with the published article is incorrect. The correct Data Availability Statement should read: Data are available from Dryad Digital Repository (doi:10.5061/dryad.r7865).
