# Supplementary material for: Patients’ Experiences with and Attitudes towards a Diabetes Patient Web Portal
Source: PLoS One. 2015 Jun 18;10(6):e0129403. doi: 10.1371/journal.pone.0129403 (PMC4472519; doi:10.1371/journal.pone.0129403)
Supplement: S1 Questionnaire — (DOCX) [file pone.0129403.s001.docx]

**Questionnaire**

**1. How did you became aware of the Patient Web Portal?**

*(several answers possible)*

a. My physician, diabetes nurse or nurse practitioner told me about it.

b. I saw a poster about the Patient Web Portal.

c. I received an informational pamphlet about the Patient Web Portal.

d. By means of friend/family who uses it him/herself.

e. Other.

**2. What is the reason you decided to start using the Patient Web Portal?**

*(several answers possible)*

a. Because it would give me influence on disease and management

b. Because it be convenient to have access to all my data (clinic notes and test results) at home

c. because others thought it would be useful for me

d. Because I was discontent with the current care I receive

e. Other.

**3. How often do you access the Patient Web Portal?**

a. daily

b. at least once a week

c. at least once a month

d. less than once a month

**4. How long do you usually access the Patient Web Portal?**

a. less than fifteen minutes

b. less than thirty minutes

c. less than one hour

d. over an hour

**5. If you have ever needed the helpdesk, what was the reason for this?**

a. I have never needed the helpdesk

b. I wanted to receive information about the procedure for requesting a login

c. I lost my username and/or password

d. I had trouble finding the information I wanted within the Patient Web Portal

e. I had trouble imputing data in the Patient Web Portal

f. Other

**6. How often do you access the Internet other than the Patient Web Portal?**

a. daily

b. at least once a week

c. at least once a month

d. never *(skip the next question)*

**7. How long do you usually access the Internet per session?**

a. less than fifteen minutes

b. less than thirty minutes

c. less than one hour

d. over one hour

**8. What is the reason you use the Internet?**

*(several answers possible)*

a. For my work

b. For socializing (e.g. email, hyves, facebook)

c. For looking up information (e.g. on diabetes)

d. For relaxation (e.g. videogames)

e. Other

**9. Do you use the Internet for looking up diabetes information?**

a. daily

b. at least once a week

c. at least once a month

d. never

**10. Is the information provided in the Patient Web Portal comprehensible for you, in specific the**

(*yes/no)*

a. meaning of laboratory values

b. abbreviations

c. medical terminology

d. reason behind the check-ups by different providers

**11. Can you indicate the importance of the following features of the Patient Web Portal?**

*(Scored on 5-point Likert scale ranging from “very useful” to “not at all useful”)*

1. Summary of upcoming visits
2. Summary of all physicians/caregivers
3. The possibility of e-messaging
4. Access to general diabetes information
5. Possibility of tracking glucose in the glucose diary
6. Rereading clinic notes
7. Access to personal laboratory values and treatment goals (e.g. blood test and weight)
8. Summary of all controls (past en future)
9. Summary of your medication

**12. How satisfied are you with the Patient Web portal with regard to…**

*(scored on a 5-point Likert scale ranging from “very satisfied” to “not at all satisfied”)*

1. How easy it is to use
2. Layout, letter type and color
3. How easy it is to login
4. Comprehensibility of the overall information displayed (such as background information about diabetes and all your own data)
5. Helpdesk *(only answer this if you ever contacted the helpdesk)*

**13. Do you know the value of your weight?** *(yes/no)*

**14. Do you know the value of your blood pressure?** *(yes/no)*

**15. Do you know of the value of your HbA1c?** *(yes/no)*

**16. Do you know the value of your cholesterol?** *(yes/no)*

**17. Do you know the treatment goals of your** *(yes/no)*…

a. …weight?

b. …blood pressure?

c. …HbA1c?

d. …cholesterol?

**18. Do you believe the Patient Web Portal will help with…**

*(yes/no/not applicable)*

1. Adherence to diet
2. Adherence to sport
3. Losing weight
4. Stop smoking
5. Adherence in taking medication
6. Improving diabetes knowledge
7. Preventing complications (such as low blood sugar and feet problems)

**19. The following questions are about the future of the Patient Web Portal. We would like to understand if you would be interested in the following possible additional features:**

*(scored on a 5-point Likert Scale ranging from “very important” to “not at all important”)*

1. Receiving an automatic message (reminder) of a clinic visits by means of email or sms
2. Sending automatic message to the physician when my uploaded glucose levels are too high or too low
3. Automatic upload from my glucose meter to the glucose diary in the PWP
4. The possibility of adding the injected insulin units to the glucose diary *(only answer this question if you use insulin)*
5. Referral to websites with more diabetes information (such as diep.info or a patient association)
6. Referral to websites that can actively help me met my goals (such as quit smoking or dieting)
7. Possibility of logging on the Patient Web Portal on the mobile phone (through app)
8. Request for medication refills
9. Forum functionality (for contact with peers)
10. Print functionality
11. Newsfeed with the latest news on diabetes
12. Possibility to look at the information in a different language (e.g. Turkish, Moroccan)
13. More information about the reason why I use the which medications and what the side-effects of these medications are
14. Scheduling a clinic visit with physician
